# Sequential Surgical Management of Refractory Primary Angle-Closure Glaucoma in a Functionally Monocular Patient

**DOI:** 10.3390/life16060907

**Published:** 2026-05-28

**Authors:** Valeria Coviltir, Maria Cristina Marinescu, Miruna Gabriela Burcel, Cosmin Adrian Teodoru, Maria-Emilia Cerghedean-Florea

**Affiliations:** 1 Ophthalmology Discipline, Carol Davila University of Medicine and Pharmacy, 8 Eroii Sanitari Blvd, 050474 Bucharest, Romania; 2 Department of Ophthalmology, Clinical Institute of Ophthalmological Emergencies “Prof. Dr. Mircea Olteanu”, 010464 Bucharest, Romania; 3 Medical Physiology Discipline, Carol Davila University of Medicine and Pharmacy, 8 Eroii Sanitari Blvd, 050474 Bucharest, Romania; 4 Faculty of Medicine, Transilvania University of Brasov, 56 Nicolae Bălcescu Street, 500019 Brasov, Romania; 5 Brasov County Emergency Clinical Hospital, 25 București Avenue, 500326 Brasov, Romania; 6 Faculty of Medicine, “Lucian Blaga” University of Sibiu, 550024 Sibiu, Romania

**Keywords:** primary angle-closure glaucoma, hyphema, vitreous hemorrhage, phacotrabeculectomy, Ahmed valve

## Abstract

Background: Primary angle-closure glaucoma (PACG) carries a high risk of irreversible blindness. This case highlights the challenges of managing refractory PACG in a patient with a history of prematurity and the subsequent development of postoperative complications. Case Presentation: A 45-year-old woman with a history of prematurity and monocular vision (no light perception in left eye) presented with acute angle closure in the right eye (RE). Despite maximal medical therapy, the RE intraocular pressure (IOP) remained at 54 mmHg. Less invasive options like laser iridotomy or standalone lens extraction were deemed insufficient, and to achieve efficient IOP lowering, the patient underwent combined phacotrabeculectomy, which was soon complicated by malignant glaucoma (aqueous misdirection) and hyphema. This necessitated a secondary intervention involving pars plana anterior vitrectomy and posterior capsulotomy. Over an 18-month follow-up, the patient suffered recurrent complications, including a spontaneous vitreous hemorrhage and eventually uncontrolled IOP. Management required a vitrectomy and the subsequent implantation of an Ahmed glaucoma valve. Although the valve procedure was followed by a transient early postoperative hyphema, the patient’s condition stabilized. At the final 28-month follow-up, the RE best corrected visual acuity was 20/25 with an IOP of 12–18 mmHg without medication. Conclusions: This case notes an interesting coexistence of advanced adult-onset angle-closure glaucoma and a history of prematurity, though a direct causal or anatomical link remains speculative due to the lack of advanced anterior segment imaging. The clinical course underscores the necessity of aggressive surgical management for aqueous misdirection and the risk of recurrent intraocular hemorrhage in advanced glaucoma. While a definitive developmental relationship cannot be established from a single case, this presentation highlights that an individualized history of prematurity may be a noteworthy consideration during comprehensive long-term ophthalmic evaluations.

## 1. Introduction

Glaucoma is the most common etiology of irreversible blindness worldwide, leading to visual impairment in 5.7 million people worldwide [1] specifically affecting the retinal ganglion cells, nerve fiber layers, and visual field, often involving raised intraocular pressure (IOP) [2]. Of the two types of primary glaucoma types, the primary angle-closure glaucoma often has a more dramatic outcome, with a 25% lifetime risk of blindness for the patient [3]. We report the longitudinal surgical management of a functionally monocular patient with refractory primary angle-closure glaucoma complicated by malignant glaucoma and recurrent hemorrhagic events.

## 2. Case Presentation

A 45-year-old woman presented through the Emergency Department to our clinic for significant pain and decrease in visual acuity (VA) in the right eye (RE) and a frontal headache. The patient’s family history was unremarkable. Her birth and developmental history was notable for prematurity via Cesarean section delivery due to placenta previa. She had a low birth weight of 1300 g and was small for gestational age; the neonatal ophthalmic evaluation indicated the absence of retinopathy of prematurity (ROP).

The patient’s medical history included high blood pressure, chronic gastritis, complicated birth at 29 years old, with hemorrhagic shock, followed by 9 days of coma. Ophthalmological history is relevant for significant myopic shift in the left eye (LE) at age 25 (spherical equivalent from −1.00 Dsph to −8.00 Dsph), LE acute angle closure at age 30, treated with topical and systemic hypotensive medication, followed by surgery (trabeculectomy, lens extraction with aphakia), with poor outcome—VA LE postoperative was no light perception (NLP). Following this episode, she was also diagnosed with primary angle-closure glaucoma in the right eye, and she used topical medication in both eyes (BE), first β-Blocker (βB) twice daily, followed by fixed combination prostaglandin analogue and βB (PG + βB) once daily, finally reaching intraocular pressure (IOP) target with PG once daily and fixed combination βB and carbonic anhydrase inhibitor (βB + CAI) twice daily. According to the patient, she did not use pilocarpine as she did not tolerate the side effects.

**Figure 1:**
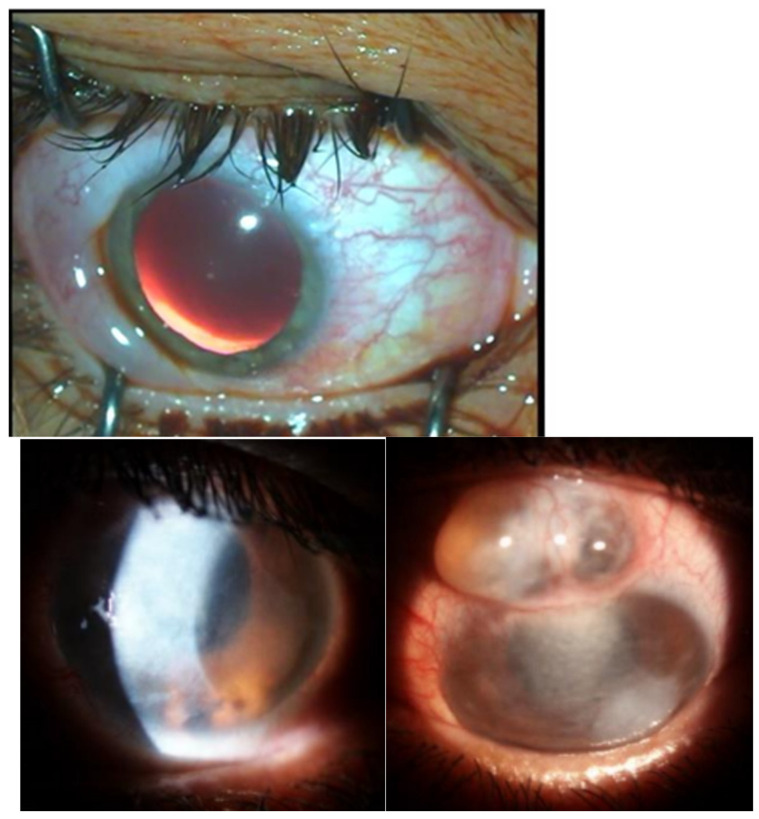
Slit-lamp aspect of right eye (**first image**) and left eye (**second** and **third image**) at presentation.

On admission, the ophthalmological examination revealed best-corrected visual acuity (BCVA) in RE 20/200 (Snellen chart), in LE NLP. IOP was 50 mmHg in the RE, 26 mmHg in the LE; BE under PG once daily and βB + CAI twice daily, and oral acetazolamide, 250 mg, twice daily. Central corneal thickness in the RE was 484 micrometers (adjusted RE IOP is 54 mmHg). Slit lamp examination showed RE epithelial and stromal corneal edema, shallow anterior chamber (AC), non-reactive mid-dilated pupil, cortical and nuclear lens opacities; LE exotropia, diffuse corneal opacity, deep AC with pigment dispersion, peripheral iridectomy at 10 and 1 o’clock, cystic filtering bleb, iridodonesis, fixed mid-dilated pupil, and surgical aphakia (see Figure 1).

Gonioscopy could only be performed in the RE, with difficulty, revealing iridotrabecular contact in all quadrants. Fundus examination could only be performed in the RE, with difficulty due to opaque media, revealing a well delineated optic nerve head, with significant cupping (vertical cup-to-disc ratio of 0.8–0.9), unremarkable macular region, mild generalized retinal arteriolar narrowing.

**Figure 2:**
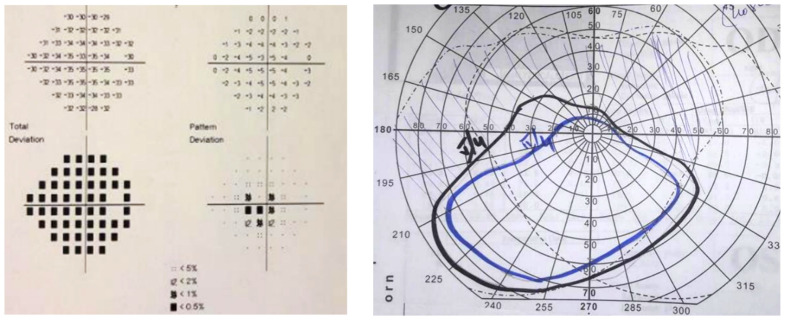
(**Left image**): Automated perimetry examination of the right eye revealed marked decrease in general sensitivity, with poor fixation (MD −33.13 dB, PSD 1.71 dB). (**Right image**): Goldmann kinetic perimetry demonstrating marked concentric constriction of the visual field (field loss is most pronounced in the inferior and temporal meridians), with more accentuated constriction for a smaller light stimulus (blue isopter). The left eye had no point of fixation, so investigation could not be performed.

Diagnostic testing included visual field testing for the RE, performed both manually and automated, which revealed a marked general reduction in sensitivity (see Figure 2), ocular echography for the LE, revealing clear vitreous and attached retina. Furthermore, the optical biometry of the RE measured axial length (AL) of 22.14 mm, anterior chamber depth (ACD) of 2.23 mm, keratometry of 46.17 D/47.47 D. Unfortunately, lens thickness could not be measured, and ultrasound biomicroscopy (UBM) or anterior-segment optic coherence tomography (AS-OCT) were also unavailable. Due to opaque media, OCT could not be performed in order to assess glaucomatous neuropathy (retinal nerve fiber layer, ganglion cell complex).

Corroborating the clinical and paraclinical data, the diagnosis was RE Acute angle closure, primary angle closure glaucoma, corticonuclear cataract, hypertensive retinopathy stage I and LE absolute glaucoma, surgical aphakia, bullous keratopathy. The patient received systemic and topical hypotensive medication, with only partial improvement (RE IOP to 42 mmHg) and underwent combined surgery, phacoemulsification with intraocular lens (IOL) implantation in the posterior chamber, trabeculectomy and peripheral iridectomy.

The immediate postoperative course was initially favorable, with RE IOP of 20 mmHg and slit lamp examination revealing conjunctival hyperemia, diffuse subconjunctival filtering bleb, clear cornea, shallow AC, pharmacologic mydriasis, patent superior PI, well positioned IOL. However, the second day the RE VA decreased to hand motion (HM), IOP increased to 30 mmHg, anterior segment showed marked hyperemia, flat filtering bleb, epithelial and stromal corneal edema, very shallow AC, hyphema in the pupillary area and within the iris crypts, non-reactive semimydriasis, with patent iridectomy and well-placed IOL. The patient underwent systemic hypotensive treatment with intravenous mannitol. However, the next day the hyphema was stationary, the automated perimetry revealed the absence of a fixation point, ocular echography showed vitreous opacities with medium-high reflectivity, attached retina (thereby ruling out choroidal detachment and suprachoroidal hemorrhage). The patient was diagnosed with malignant glaucoma (aqueous misdirection), as an early postoperative complication. Surgical intervention performed in the third postoperative day consisted of anterior chamber washout and blood aspiration, pars plana anterior vitrectomy with posterior capsulotomy, anterior chamber reformation using methylcellulose, and intravitreal administration of anti-VEGF therapy. The anti-VEGF agent was used based on the potential benefit of blood–aqueous barrier stabilization and, therefore, its effect on hyphema [4].

Following this second intervention, the RE BCVA increased to 20/40, IOP was controlled at 20 mmHg without medication, anterior chamber and vitreous chamber were free of blood, and the patient was discharged, with periodic examinations at her local clinic, which demonstrated stable VA. In the following period, IOP remained under 20 mmHg; however, the treatment burden increased stepwise through monthly check-ups: after 3 months a PG daily drop was added, and 6 months later a βB twice daily drop was added, and 3 months later the final regimen was established: PG once daily and βB + CAI fixed combination twice daily.

**Figure 3:**
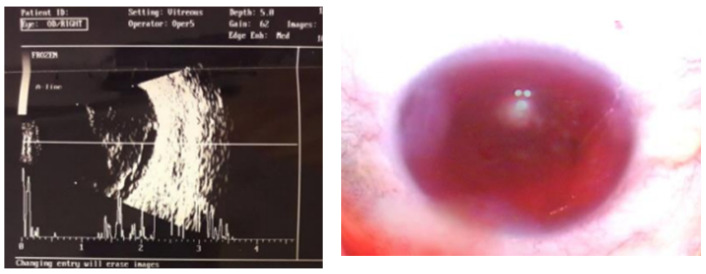
(**Left image**): Right eye echography, showing vitreous hemorrhage and epiretinal membranes. (**Right image**): Slit-lamp aspect of anterior segment of RE, showcasing significant hyphema.

Eighteen months after the first presentation, the patient returned to the Emergency department for RE sudden loss of vision. RE BCVA was HM, RE IOP was 28 mmHg, under PG and βB + CAI, slit lamp examination revealed conjunctival hyperemia, diffuse filtering bleb, narrow AC, hyphema and vitreous hemorrhage and epiretinal membranes were detected using echography (see Figure 3). The diagnosis was vitreous hemorrhage and the patient was referred to the Vitreoretinal department, where she underwent vitrectomy, epiretinal membrane excision, fluid-serum-air exchange, without intraoperative complications and with favorable postoperative evolution.

The subsequent 3 months were uneventful, with well controlled IOP under maximal topical medication, PG once daily, (βB + CAI) twice daily, α2-adrenergic once daily. However, she was referred to our clinic the next month for further glaucoma treatment. BCVA RE was 20/30, RE IOP was 30 mmHg under above-mentioned treatment, slit lamp revealing filtering bleb, well-formed AC, sluggish pupillary response, and fundus examination revealed cup-to-disc ratio of 0.9. The patient underwent RE Ahmed valve implantation in the superior temporal (ST) quadrant, without intraoperative complications—22 months after first presentation. The anterior placement of the tube was chosen as both anterior chamber angle and ciliary sulcus placement provide similar IOP decrease [5]. Firstly, the previous anterior vitrectomy, phacoemulsification and posterior capsulotomy led to a significantly deepened chamber, and anterior placement was considered safe in regard to potential endothelial cell loss [6], and secondly, posterior placement was decided against in order to avoid potential iris chafing, mechanical uveitis, or recurrent uveitis–glaucoma–hyphema syndrome [6,7], as patient already presented hemorrhagic complications.

On the first day, BCVA RE was HM, RE IOP was 22 mmHg, RE anterior segment presented chemosis, palpebral ecchymosis, ST subconjunctival Ahmed valve, corneal edema, shallow AC with hyphema. Ocular echography revealed vitreous opacities with low-medium reflectivity, attached retina. The hyphema remitted due to treatment with intravenous mannitol, vitamin C, ethamsylate and local antibiotic and non-steroidal anti-inflammatory, NSAID (local RE), BCVA increased and IOP normalized; thus the patient was discharged.

**Figure 4:**
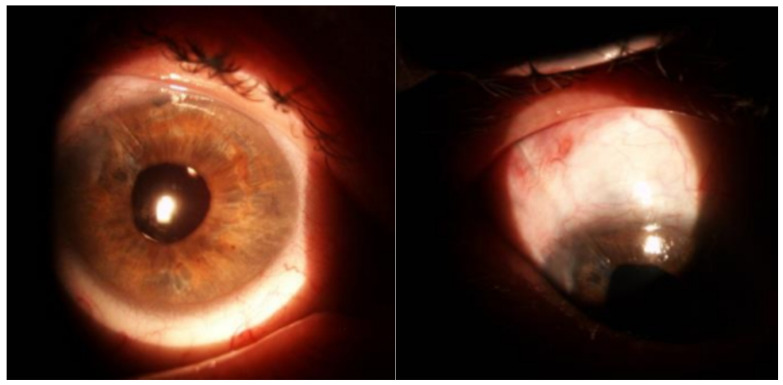

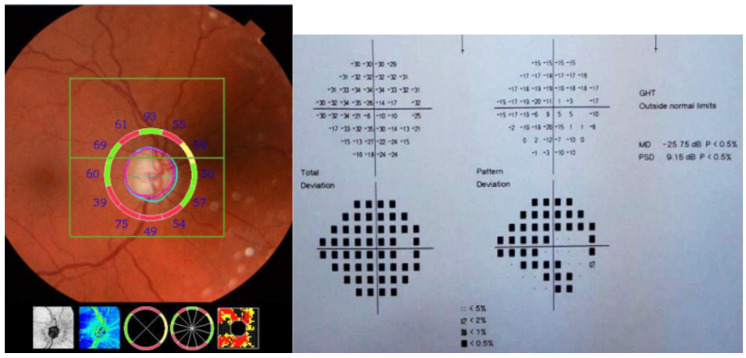
(**Upper right** and **left images**): final slit lamp aspect of the RE. (**Lower left**): Fundus photography of RE, showcasing c/d ratio of 0.9, together with decreased inferior and superior retinal nerve fiber layer (RNFL) thickness. (**Lower right**): Automated perimetry, revealing markedly decreased retinal sensitivity and a paracentral island of vision.

Subsequent visits involved monthly check-ups, with favorable evolution. RE BCVA increased to 20/25, IOP remained stable between 12 and 18 mmHg, without topical medication, anterior segment presented with ST subconjunctival Ahmed valve, with the tube in the AC, diffuse filtering bleb, clear cornea, well-formed AC, superior peripheral iridectomy, round central reactive pupil, well-positioned IOL, posterior capsulotomy, and fundus presented a cup-to-disc ratio of 0.9 and generalized retinal arteriolar narrowing. The automated perimetry in the RE has also improved, with an MD of −25.75 dB and a paracentral island of vision (Figure 4). An overview of the clinical evolution can be found in Table 1.

## 3. Discussion

The present case poses several questions and challenges—throughout the complicated evolution of maintaining usable vision in a functionally monocular patient, one must focus on the particularity of malignant glaucoma after phacotrabeculectomy in angle-closure disease and, thus, anatomic predisposition, and also on the surprising postoperative hemorrhagic complications. Another important risk for an eye undergoing repeated surgical interventions and long-term topical hypotensive treatment is corneal decompensation and significant ocular surface alterations, and while tear film component imbalance and inflammatory mediator dysregulation have been associated with impaired ocular surface homeostasis, the present patient maintained good corneal clarity throughout the clinical course [8].

The initial surgical choice in this case was combined phacotrabeculectomy, despite the negative evolution of a similar surgical approach in the other eye. Other choices considered by the surgical team were peripheral laser iridotomy and phacoemulsification—the two options considered in the landmark EAGLE study [9]. However, the EAGLE trial included patients without cataract and an average IOP of 30 mmHg. As such, it would have excluded a patient such as ours, with visually significant cataract and extremely high IOP of 54 mmHg, and lens extraction or laser iridectomy would not have provided the aggressive IOP decrease needed in this context. Furthermore, the European Glaucoma Society’s most recent Guidelines support the addition of filtration surgery to lens extraction in severe disease, in patients with PACG and cataract, while also admitting that a low axial length acts as a risk factor for aqueous misdirection, as occurred in our patient [10].

One of the most feared complications of the trabeculectomy intervention is aqueous misdirection (more commonly called malignant glaucoma), and comprises a forward movement of the lens-iris diaphragm, secretion of aqueous into the vitreous cavity and, ultimately, angle closure [10]. In our patient, the elements supporting this diagnosis were firstly a very shallow anterior chamber, together with high IOP and a flat filtering bleb [11]. Other potential diagnoses were eliminated: the patent iridectomy eliminated the possibility of pupillary block, and overfiltration was unlikely in the presence of high IOP [11]. Anatomical risk factors for malignant glaucoma were present in our patient, namely the preexisting shallow chamber [12], and patients with high IOP are more likely to require surgical intervention, namely pars plana vitrectomy [13].

Importantly, the patient did not receive topical pilocarpine throughout this follow-up period, as it may move forward the iris-lens diaphragm, further shallowing the anterior chamber, particularly in lens-induced cases, or cases in which the closure is worsened by the lens, such as our patient [10].

Further, while some research suggests a potential connection between prematurity and adult-onset ocular pathologies like early-age cataracts or angle-closure glaucoma [14,15], any such association in our patient remains purely hypothetical. An additional particularity of the present case is the coexistence of progressive myopic shift and severe angle-closure disease—the latter being associated with hyperopia [16,17,18]. Given that neither UBM nor AS-OCT was available to evaluate the lens and ciliary body architecture, a lenticular mechanism—such as refractive index change or anterior lens rotation—can only be proposed as a remote possibility rather than a definitive conclusion, and an explicit etiologic link in our patient remains unverified. Similarly, while a developmental anterior segment discontinuity leading to relative anterior microphthalmia has been described in the literature, we cannot confirm if this mechanism contributed to our patient’s severe angle-closure disease [19].

Another notable aspect of this case was the recurrent hemorrhagic postoperative evolution, including early postoperative hyphema following both phacotrabeculectomy and Ahmed valve implantation and vitreous hemorrhage during long-term follow-up. Early postoperative hyphema is a relatively common complication, reported in 3.9–63% of trabeculectomies [20]. Although the patient did not exhibit the major risk factors for this complication—anticoagulant or antiplatelet treatment, and neovascular glaucoma—they did present other important risk factors—high preoperative IOP [20] and low postoperative IOP [21]. Further, the patient denied any trauma or systemic bleeding disorders, and slit-lamp examination excluded any IOL- or retina-related causes, or the existence of neovessels anywhere. The vitreous hemorrhage and hyphema, not precipitated by recent trauma or ocular surgery, may have involved several potential mechanisms: the first glaucoma intervention, followed by hyphema and reintervention, may have led to neovessels at the surgical wound (resembling Swan syndrome—in which an ingrowth of episcleral vessels at the cataract wound site develop and associate recurring intraocular bleeding), with a high risk of spontaneous hemorrhage into the vitrectomized anterior vitreous cavity [22,23]. However, follow-up gonioscopies failed to identify the sclerostomy neovessel, and UBM was not available to investigate potential IOL-related bleeding (uveitis–glaucoma–hyphema plus syndrome, which also involves vitreous hemorrhage) [22,24,25]. Alternatively, the patient’s systemic hypertension and advanced chronic glaucoma may have heightened ocular vascular fragility, making the microvasculature more vulnerable to structural damage during periods of elevated IOP [26,27].

## 4. Conclusions

In conclusion, while this complex case demonstrates a successful long-term surgical outcome for refractory PACG, the potential link between prematurity-associated developmental alterations and adult-onset angle closure remains observational and hypothetical. This case highlights an intriguing clinical presentation; albeit it is difficult to generalize and suggest a certain clinical approach. Instead, it suggests that an individualized approach to long-term monitoring may be beneficial when managing patients with a history of prematurity who exhibit atypical ocular anatomy or early-onset progressive changes.

In terms of surgical approach, such a refractory glaucoma proves to be challenging, with multiple interventions needed for disease control and a reserved long-term prognosis. Lastly, the patient’s quality of life, especially in case of advanced glaucoma, is severely impaired, due to the diagnosis of a chronic disease which may lead to blindness and which involves lifelong dependence on a treatment, with possible side effects, financial costs, potential future loss of visual field.

## Figures and Tables

**Table 1:** Complete timeline of clinical case evolution.

Time	Clinical Findings	BCVA	IOP	Intervention	Outcome
Birth (premature)	No ROP detected			Neonatal ophthalmologic evaluation	Normal retinal findings
Age 25	LE progressive myopic shift	20/20 with −8.00 Dsph		Refractive correction	Increasing anisometropia
Age 30	LE acute angle closure	Severe visual loss		Trabeculectomy + lens extraction	LE NLP
	RE PACG	RE adequate vision (exact values unavailable), LE NLP	RE high IOP, LE IOP control post surgery	PG + βB + CAI	Temporary RE IOP control
Age 45 (first presentation to current clinic)	RE Acute angle closure, corticonuclear cataract	RE 20/200	RE 50 mmHg	Systemic + topical hypotensives	Partial stabilization
		RE 20/200	RE 42 mmHg	Phacotrabeculectomy + Peripheral Iridectomy	Early postoperative improvement
Postoperative day I	RE Status post phaco-trabeculectomy		RE 20 mmHg		
Postoperative day II	Shallow AC, hyphema	RE HM	IOP 30 mmHg	Diagnosed malignant glaucoma (aqueous misdirection syndrome)	Surgical indication
Second surgery (post-op day III)	Aqueous misdirection			PP anterior vitrectomy + posterior capsulotomy	BCVA 20/40
Age 47 (18 months after the first presentation)	Vitreous hemorrhage	RE HM	RE 28 mmHg	Pars plana vitrectomy	Favorable evolution
Age 47 (Next follow-up—22 months after first presentation)	Uncontrolled glaucoma	RE 20/30	RE 30 mmHg	Ahmed valve implantation	IOP control
Age 48 (Final follow-up—28 months after first presentation)	Stable advanced glaucoma	RE 20/25	RE 12–18 mmHg	Observation	Monocular vision preserved

## Data Availability

No new data were created or analyzed in this study. Data sharing is not applicable to this article.

## Abbreviations

The following abbreviations are used in this manuscript:

AC	Anterior chamber
ACD	Anterior chamber depth
AL	Axial length
AS-OCT	Anterior-segment optical coherence tomography
BCVA	Best-corrected visual acuity
BE	Both eyes
CAI	Carbonic anhydrase inhibitor
Dsph	Spherical diopters
HM	Hand motion
IOL	Intraocular lens
IOP	Intraocular pressure
LE	Left eye
MD	Mean deviation
NLP	No light perception
NSAID	Non-steroidal anti-inflammatory drug
OCT	Optical coherence tomography
PACG	Primary angle-closure glaucoma
PG	Prostaglandin analogue
PI	Peripheral iridectomy
PP	Pars plana
PSD	Pattern standard deviation
RE	Right eye
RNFL	Retinal nerve fiber layer
ROP	Retinopathy of Prematurity
ST	Superior temporal
UBM	Ultrasound biomicroscopy
VA	Visual acuity
VEGF	Vascular endothelial growth factor

## References

[1] Nicola C.A., Marinescu M.C., Firan A.M., Tartea G., Naidin M.S., Ciuluvica R.C., Dimulescu M.D., Voicu N.M., Mihailescu C.M., Meca A.-D. (2025). Changes in Quality of Life Among Glaucoma Patients Following Six Months of Niacinamide Supplementation. Nutrients.

[2] Potop V., Coviltir V., Corbu C., Burcel M.G., Ionescu C.I., Dascalescu D.M.C. (2019). Corneal Hysteresis, A Glaucoma Risk Factor Independent Of The Intraocular Pressure. Rev. Roum. Des. Sci. Tech. Série Électrotech. Énergétique.

[3] Potop V., Dragosloveanu C.D.M., Ciocâlteu A.M., Burcel M.G., Marinescu M.C., Dăscălescu D.M.C. (2024). The Mirror Theory: Parallels between Open Angle and Angle Closure Glaucoma. Life.

[4] Song X., Li L., Wang X., Zhang X., Lei Q., Liu G., Wang L., Chen J. (2025). Intravitreal Anti-Vascular Endothelial Growth Factor Agents as an Adjunct for Proliferative Diabetic Retinopathy: A Systematic Review and Meta-Analysis. BMC Ophthalmol..

[5] Bayer A., Önol M. (2016). Clinical Outcomes of Ahmed Glaucoma Valve in Anterior Chamber versus Ciliary Sulcus. Eye.

[6] Zhang Q., Liu Y., Thanapaisal S., Oatts J., Luo Y., Ying G.-S., Wang J., McLeod S.D., Gedde S.J., Han Y. (2021). The Effect of Tube Location on Corneal Endothelial Cells in Patients with Ahmed Glaucoma Valve. Ophthalmology.

[7] Altwijri R.J., Alsirhy E. (2024). Uveitis Glaucoma Hyphema Syndrome as a Result of Glaucoma Implant: A Case Report. World J. Clin. Cases.

[8] Burcel M.G., Constantin M., Ionita G., Dascalescu D., Ionescu C., Stanila D., Potop V., Coviltir V. (2020). Levels of Lactoferrin, Lysozyme and Albumin in the Tear Film of Keratoconus Patients and Their Correlations with Important Parameters of the Disease. Rev. Romana Med. Lab..

[9] Azuara-Blanco A., Burr J., Ramsay C., Cooper D., Foster P.J., Friedman D.S., Scotland G., Javanbakht M., Cochrane C., Norrie J. (2016). Effectiveness of Early Lens Extraction for the Treatment of Primary Angle-Closure Glaucoma (EAGLE): A Randomised Controlled Trial. Lancet.

[10] Pazos M., Traverso C.E., Viswanathan A., European Glaucoma Society, Guidelines Task Force, Guidelines Writers, Authors and Contributors, Guidelines Internal Reviewers, Experts by Experience Group (Patients’ Panel), Team of Clinica Oculistica of the University of Genoa for Medical Editing and Graphics, External Reviews (2025). European Glaucoma Society—Terminology and Guidelines for Glaucoma, 6th Edition. Br. J. Ophthalmol..

[11] Stephey E. (2024). Book Review: Kanski’s Clinical Ophthalmology: A Systematic Approach, 10th Ed. Optom. Vis. Sci..

[12] Liu Q., Shen Z., Guo L., Li D., Zhang R., Chen J., Luo P., Feng Z., Yang L. (2026). Preoperative Anterior Segment Risk Factors for Malignant Glaucoma Following Surgery in Primary Angle-Closure Glaucoma: A Retrospective Case–Control Study. J. Ophthalmol..

[13] Fujita A., Kearney W.C., Friedman D.S., Parikh P., Kelly E.C., Shweikh Y., Ross C., Elze T., Lorch A.C., Miller J.W. (2026). Causes and Treatments of Malignant Glaucoma in the United States: Analysis of IRIS^®^ Registry. Ophthalmol. Glaucoma.

[14] Mundey K., Chaudhry M., Sethi S. (2015). Long Term Ophthalmic Sequelae of Prematurity. J. Clin. Ophthalmol. Res..

[15] Fledelius H.C., Jensen H. (2011). Late Subsequent Ocular Morbidity in Retinopathy of Prematurity Patients, with Emphasis on Visual Loss Caused by Insidious “Involutive” Pathology: An Observational Series. Acta Ophthalmol..

[16] Chen T.-C., Tsai T.-H., Shih Y.-F., Yeh P.-T., Yang C.-H., Hu F.-C., Lin L.L.-K., Yang C.-M. (2010). Long-Term Evaluation of Refractive Status and Optical Components in Eyes of Children Born Prematurely. Investig. Ophthalmol. Vis. Sci..

[17] Larsson E.K., Rydberg A.C., Holmström G.E. (2003). A Population-Based Study of the Refractive Outcome in 10-Year-Old Preterm and Full-Term Children. Arch. Ophthalmol..

[18] Marinescu M.-C., Dascalescu D.-M.-C., Constantin M.-M., Coviltir V., Potop V., Stanila D., Constantin F., Alexandrescu C., Ciuluvica R.-C., Voinea L.-M. (2023). Particular Anatomy of the Hyperopic Eye and Potential Clinical Implications. Medicina.

[19] Yong K.-L., Gong T., Nongpiur M.E., How A.C., Lee H.K., Cheng L., Perera S.A., Aung T. (2014). Myopia in Asian Subjects with Primary Angle Closure: Implications for Glaucoma Trends in East Asia. Ophthalmology.

[20] Kojima S., Inatani M., Shobayashi K., Haga A., Inoue T., Tanihara H. (2014). Risk Factors for Hyphema after Trabeculectomy with Mitomycin C. J. Glaucoma.

[21] Iwasaki K., Katsuo A., Arimura S., Takamura Y., Inatani M. (2026). Risk Factors for Postoperative Hyphema Following Baerveldt Glaucoma Implant Surgery: A Retrospective Cohort Study. J. Clin. Med..

[22] Leroux P., Rezkallah A., Kodjikian L., Loria O., Mathis T., Denis P. (2021). Recurrent Hyphema after Trabeculectomy: An Atypical Case of Swan Syndrome. Int. J. Ophthalmol..

[23] Carrasquillo A.M., Gupta B.K., Wilensky J.T. (2001). Recurrent Hyphema in an Aphakic Child: Swan Syndrome. J. Am. Assoc. Pediatr. Ophthalmol. Strabismus.

[24] Alniemi S.T., Amin S.R., Sculley L., Bakri S.J. (2018). Ultrasound Biomicroscopy in Pseudophakic Patients with Unexplained Recurrent Hyphema or Vitreous Hemorrhage. Semin. Ophthalmol..

[25] Sousa D.C., Leal I., Faria M.Y., Pinto L.A. (2016). A Rare Manifestation of Uveitis-Glaucoma-Hyphema Syndrome. J. Curr. Glaucoma Pr..

[26] Tripathy K., Arsiwalla T. (2025). Hypertensive Retinopathy. StatPearls [Internet].

[27] Wang X., Wang M., Liu H., Mercieca K., Prinz J., Feng Y., Prokosch V. (2023). The Association between Vascular Abnormalities and Glaucoma-What Comes First?. Int. J. Mol. Sci..

